# Local reports of climate change impacts in Sierra Nevada, Spain: sociodemographic and geographical patterns

**DOI:** 10.1007/s10113-022-01981-5

**Published:** 2022-12-16

**Authors:** David García-del-Amo, Peter Graham Mortyn, Victoria Reyes-García

**Affiliations:** 1 Institut de Ciència I Tecnologia Ambientals, Universitat Autònoma de Barcelona, Columnes S/N. Building ICTA-IPC (Z) UAB Campus, 08193 Bellaterra - Barcelona, Spain; 2 Department of Geography, Universitat Autònoma de Barcelona, 08193 Bellaterra - Barcelona, Spain; 3 Institució Catalana de Recerca I Estudis Avançats (ICREA), Barcelona, Spain

**Keywords:** Climate change impacts, Local ecological knowledge, Local perception, Mountain region

## Abstract

While we know that climate change is having different impacts on various ecosystems and regions of the world, we know less how the perception of such impacts varies within a population. In this study, we examine patterns of individual variation in climate change impacts reports using data from a sample (*n* = 238) drawn from 33 mountainous municipalities of Sierra Nevada, Spain. Sierra Nevada inhabitants report multiple climate change impacts, being the most frequently reported changes in snowfall and snow cover, abundance of terrestrial fauna, freshwater availability, and extreme temperatures. Reports of climate change impacts vary according to informants’ sociodemographic characteristics and geographical location. People with life-long bonds with the environment and higher connection and dependence upon ecosystem services report more climate change impacts than other informants, as do people with lower level of schooling. We also found that reports of climate change impacts vary according to geographic areas, which reinforces the idea that climate change generates differentiated impacts even at small geographical scales. Understanding intracultural variation in reports of climate change impacts not only gives an enriched picture of the human dimensions of climate change but might also help design more targeted mitigation and adaptation responses.

**Supplementary Information:**

The online version contains supplementary material available at 10.1007/s10113-022-01981-5.

## Introduction

Climate change is generating unevenly distributed impacts across different ecosystems and regions of the world. Thus, polar regions, low-lying coasts, deserts, small islands, and mountains, are considered hotspots of vulnerability (IPCC 2019a, b, 2021). In the same way, people with different sociodemographic characteristics are also unevenly affected by climate impacts, with communities who directly depend on natural resources for their daily subsistence being disproportionally impacted (IPBES 2019; IPCC 2022). Among the groups considered more directly affected by climate change impacts, a great deal of research attention has gone to Indigenous Peoples and local communities (IPLC).

Indigenous and local knowledge (ILK) systems, sometimes developed over hundreds of years through a history of interdependencies and co-evolution between people and nature, situate IPLC as first-hand witnesses of climate change impacts in their local environments, and this knowledge can be particularly relevant in areas where there is a lack of local instrumental data (Hock et al. 2019; Olsson et al. 2019). Several works have showed the wealth of overlapping of instrumental measurements of climate change impacts and IPLC perception of those impacts, but also that ILK systems can contribute with more detailed and new information not detected by instrumental measurements (Fassnacht et al. 2018; Hu et al. 2020; Sharafi et al. 2021; Wilson et al. 2021). Oral histories, proverbs, seasonal calendars, hunting and fishing routes, and other cultural expressions rooted on ILK can provide historical former conditions of environmental state useful to assess local climate change impacts (Janif et al. 2016; Cochran et al. 2016; Hurlbert et al. 2019; IPBES 2019; Meredith et al. 2019; Garteizgogeascoa et al. 2020). ILK is also being recognized as a fundamental source of knowledge in risk management and decision-making of environmental impacts—particularly through the involvement of IPLC to improve site-specific understanding of local climate change impacts (Hiwasaki et al. 2014; Dube and Munsaka 2018; Collins et al. 2019; Sharafi et al. 2021)—as well as in to inform policy and achieve effective social adaptation (Ford et al. 2016; Mapfumo et al. 2017; Hill et al. 2020; Petzold et al. 2020; Schlingmann et al. 2021). Indeed, since the Fifth Assessment Report (AR5) in 2014, the Intergovernmental Panel on Climate Change (IPCC) recognizes the importance of drawing on different knowledge systems to address climate change (Ford et al. 2016; David-Chavez and Gavin 2018), and voices are growing to recognize IPLC rights and agency in environmental agendas (Stringer et al. 2018; Reyes-García et al. 2021).

A major challenge to incorporate IPLC’s perspectives in climate change research and policy lies in the fact that there is not only a plurality and heterogeneity of worldviews, but also an uneven distribution of knowledge and perceptions of climate change impacts across groups. Moreover, individual sociodemographic characteristics might shape how people within a group perceive and report climate change impacts, thus generating intracultural variation on reports. Indeed, as other types of knowledge, individual reports of climate change impacts can be shaped by a diversity of factors that mediate how a person relates to the environment. Such factors range from geographical location (e.g., the area mainly used by a person) to demographic (e.g., the sex of a person) or socio-economic factors (e.g., the economic activities that the person conducts in iteration with the natural environment). Different authors have argued that understanding variation in reports of climate change impacts across people with different sociodemographic characteristics can improve climate change adaptation planning (Armah et al. 2015; Collins et al. 2019; Hurlbert et al. 2019; Petzold et al. 2020). However, there is scant research on what are the factors that pattern such variation of reports of climate change impacts.

To start filling this research gap, here we document observations of climate change impacts reported by people living in rural communities in a mountainous region of Spain and assess how participants’ sociodemographic characteristics and geographical location shape their reports. We focus on a mountain area because mountains are hotspots of climate change impacts due to the diversity of ecosystems related to their altitude, narrow zonation hydrologic importance, and extreme conditions (Palomo 2017; Zamora et al. 2017; Hock et al. 2019; Payne et al. 2020). Observed climate change impacts in mountain biological systems include vertical displacement of species towards mountaintops and declining species diversity (Bani et al. 2019; Lehikoinen et al. 2019; Hansson et al. 2021), changes in species composition (Roth et al. 2014; Duque et al. 2015), and phenological changes among others (Maikhuri et al. 2018; Prevéy et al. 2020). Additionally, we also focus on mountain areas because local populations living in mountain areas have proved to be good observers of climate change impacts. Thus, several works have shown the advantage of working with local populations on mountain areas by detecting a great variety and detailed climate change impacts in the atmospheric, physical, and biological system, but also in aspects of their livelihoods, such as a reduction of the quantity and quality of pastures (Joshi et al. 2013; Pandey et al. 2018), an increase in crop pests and livestock diseases (Postigo 2014; Taboada et al. 2017; Meldrum et al. 2018), or the extent of harvesting times and increasing crop yields (Shijin and Dahe 2015; Wang and Cao 2015).

## Methods

### Study region

We conducted research in the Sierra Nevada Natural Space (SNNS), a mountainous range with more than 172,000 hectares that runs parallel to the Mediterranean shoreline in the south-east of Spain. Given its orographic variation, SNNS presents a great variety of ecological conditions (Castillo Martín 1999; Raso Nadal 2011). The region is one of the most important European hotspots for biodiversity (Blanca et al. 1998; Pérez-Luque et al. 2015), which has resulted in its protection under the designation of Biosphere Reserve (1986), Natural Park (1989), and National Park (1999).

Given its geographical location, SNNS provides an ideal setting to study the impacts of change, for which, in 2007, the Sierra Nevada Global Change Observatory was stablished in the area (Zamora et al. 2016). Data from the Sierra Nevada Global Change Observatory shows that, since the 1970s, rainfall and snow extent and persistence have decreased (Zamora et al. 2016). Moreover, the combined effects of rising temperatures and prolonged droughts have led to shorter ice-cover periods (Pérez-Palazón et al. 2015), decreased water level, and warmer waters of high mountain lakes (Morales-Baquero et al. 2013). Reduction of snow has altered the hydrologic regime of high mountain rivers, affecting also downstream ecosystems and water users (Jódar et al. 2017). These changes have also impacted flora and fauna resulting in up-slope range shifts of insects (Menéndez et al. 2014), flora composition and distribution (Pauli et al. 2012; Fernández Calzado et al. 2012), a decrease in bird populations density, and changes in their composition. Species that were dominant in the 1980s in the lower areas of the mountain are moving up to higher levels, being replaced by other more generalist species (Zamora and Barea-Azcón 2015).

Human settlements in Sierra Nevada are documented since the Neolithic (Sánchez-Hita 2007), generating intense landscape modifications since the seventh century (Muslim period) through the construction of *acequias*, i.e., water-ditches for the management of meltwater favoring the infiltration and recharge of aquifers and increasing soil protection against erosion produced by runoff water (Jódar et al. 2018; Martos-Rosillo et al. 2019). In the middle of the twentieth century, agricultural industrialization and the rural exodus led to the abandonment of agricultural activities, a situation that, together with the reforestation with pine trees and the declaration of protected area, led to big changes in the territory (Zamora et al. 2016). Despite these changes, people working on agropastoral activities continue to rely on local knowledge and traditional management techniques for their livelihood (Iniesta-Arandia et al. 2014, García-del-Amo et al. 2022). Recent research in the region comparing the current environmental status with the former conditions reported in local sayings and proverbs (Proverbs are a short, well-known pithy saying, stating a general truth or piece of advice and are always based on the experience and observation of said facts) suggests that people are able to detect climate change impacts (Garteizgogeascoa et al. 2020).

### Sampling

**Fig. 1:**
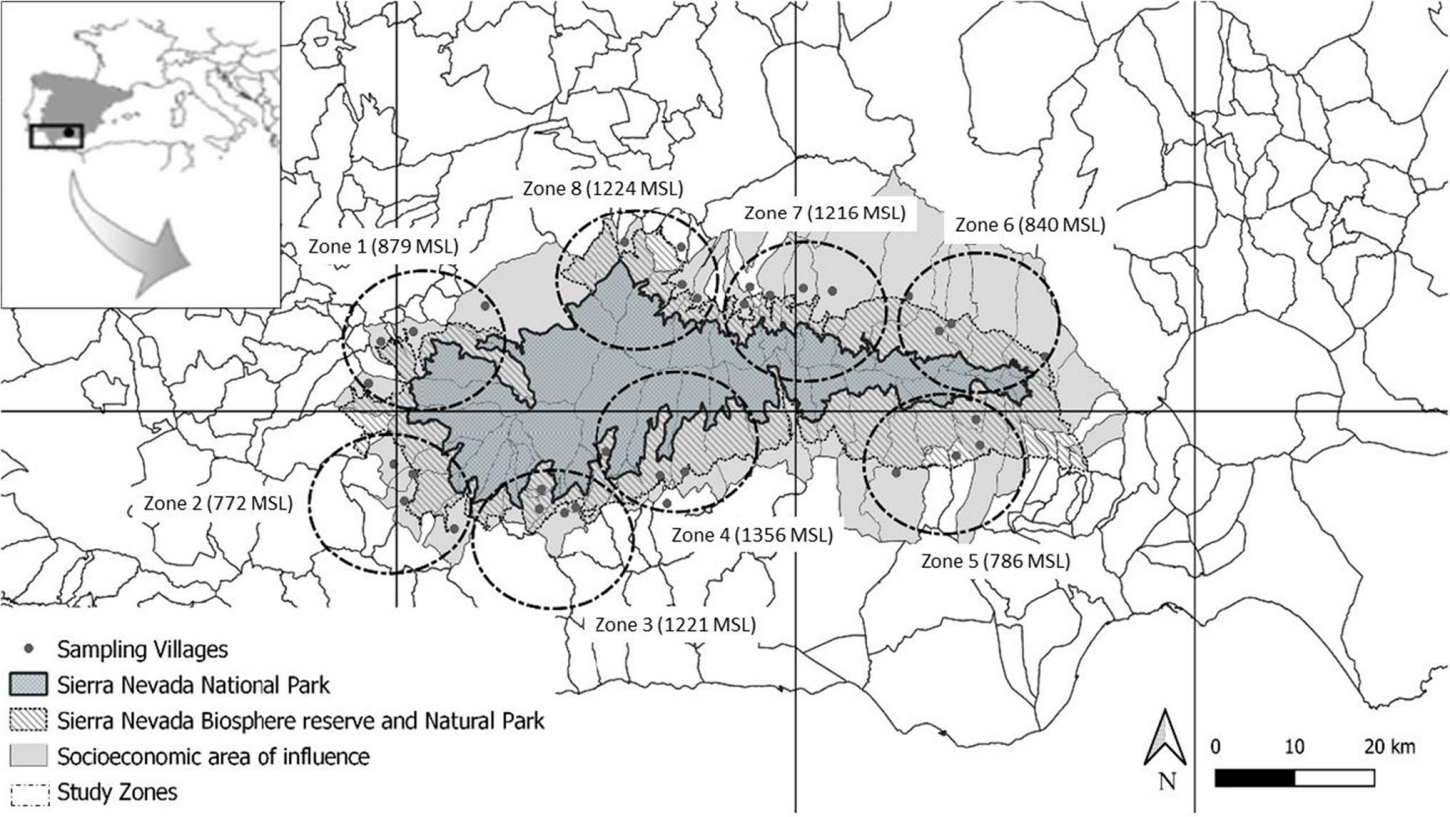
Map of the study region. Dots represent the 33 sampled villages. Circles represent the eight study Zones: Zone 1: Granada’s surroundings, Zone 2: Lanjarón and Lecrín Valley, Zone 3: Poqueira Ravine, Zone 4: Trevélez and Bérchules Ravines, Zone 5: Ohanes Ravine, Zone 6: Nacimiento Valley, Zone 7: La Calahorra Valley, Zone 8: Marquesado del Cenete. Number in brackets () represents the average of meters above sea level (MSL) of villages in a Zone

To capture potential geographical differences in climate change impacts at local scale, we conducted a purposive sampling in all villages inside the SNNS located over 500 m above sea level (MSL) and with more than 300 inhabitants. We excluded villages under 500 MSL because these villages have very different environmental conditions. We grouped the selected villages into eight Zones, each Zone encompassing 3 to 5 villages in a 10 km radius (Fig. 1).

To capture variation in sociodemographic conditions, we followed a quota sampling strategy (Shively 2011) aiming to include, in each Zone, a minimum number of individuals for each of the main professions in the area (i.e., *farmers*, *shepherds*, *ranchers*, *agricultural ranchers* (farmers who also have livestock), *beekeepers*, and *others—*including people who performed agropastoral activities as secondary economic activity or for self-consumption). Our sample only included people who have lived 25 years or longer in the village of residence and who have a profession directly or indirectly related to agropastoral activities. To select interviewees, we used different techniques. Upon arrival to a village, we held a meeting with the mayor and members of different associations to explain the research project. Through these representatives, we contacted other members of the associations in the same or other zones and asked them to participate. To find a balanced number of representatives regarding professions and to increase gender variability, we also approached potential respondents by directly visiting farms and agricultural fields located on the outskirts of the villages. Overall, we conducted 238 questionnaires in 33 villages from June to December 2018.

Our sampling and data collection procedures received the ethical approval of the Autonomous University of Barcelona (CEEAH-4781). We asked participants to sign a free, prior and informed consent form before starting data collection.

### Survey

The survey consisted of two sections: respondents’ (i) sociodemographic information and (ii) reports of climate change impacts.

In the first part of the survey, we collected information on sociodemographic characteristics of individuals including profession, age, gender, schooling, parents’ and grandparents’ origin, years of experience in agropastoral activities, and number of activities conducted in nature. Profession was coded as *farmers*, *shepherds*, *ranchers*, *beekeepers*, *agricultural ranchers*, and *others*. Information on respondent’s age was collected in years, but later grouped into age ranges of 15 years (25 to 39 years; 40 to 54 years; 55 to 69 years; 70 to 84 years; > 85 years). Respondent’s level of schooling was coded into four categories (0 = no schooling, 1 = primary school completed, 2 = secondary or vocational school completed, and 3 = bachelor or high school completed). We collected information on parent’s and grandparent’s origins in two different binary variables (0 = born outside Sierra Nevada; 1 = born in Sierra Nevada), because family origin and migration status affect individual’s local knowledge (Pirker et al. 2012; Brandt et al. 2013). Finally, we collected information on the number of activities conducted in nature as this is a good proxy for the level of interaction with the environment (Reyes-García et al. 2009). The numerical variable “number of activities in nature” (from 0 to 6) captures how many of the following activities have conducted a respondent in his/her lifetime: agriculture, livestock, beekeeping, hunting, wood collection, and non-timber forest products collection. We also noted the Zone where each informant was living. We proxied the altitude of each village as the altitude of the town hall of the village and we proxied the altitude of each Zone as the average of the altitudes of town hall of villages included in the Zone. Since villages’ lands cover a high-altitude gradient, we also created a variable, called *altitude-range*, which divides the eight Zones in four ranges (Zones with an average altitude under 800 MSL, from 800 to 1049 MSL, from 1050 to 1299 MSL, and over 1300 MSL).

In the second part of the survey, we asked informants if, when comparing the current state of the environment with the situation 25 years ago, they had noted changes. To select environmental changes to include in our survey, we relied on a literature review of local observations of climate change impacts and on semi-structured interviews. Specifically, an initial list of local observations of climate change impacts was collected from the literature and categorized in Local Indicators of Climate Change Impacts (LICCI), using the protocol proposed by Reyes-García et al. (2019). This protocol classifies observations into indicators following a hierarchical process level depending on the system, subsystem, and impacted element. We selected impacts that could have been observed in the region and confirmed their local occurrence by consulting literature on the study area and conducting interviews with 20 elderly people and 10 local experts. After conducting the interviews, we adapted the vocabulary to the local context to elaborate the survey, using local expressions like “neveros” (snowfield) or “acequias” (irrigation ditch). The final list for the survey included 95 LICCI affecting 46 impacted elements of the atmospheric (*n* = 12), the physical (*n* = 11), the biological (*n* = 15), and the human systems (*n* = 8). For each of the indicators selected, we asked respondents “Could you tell me, if you have noticed {LICCI} in this area during the last 25 years?” Responses were coded as 0 = the person did not notice the change, 1 = the person noticed the change, and 2 = person did not know (the list of LICCI used in the survey can be found in Online Resource 1). The survey was conducted in Spanish.

## Analysis

We started the analysis by exploring the impacts most commonly perceived by informants. To do so, we calculated the percentage of people perceiving each of the 95 impacts proposed in the survey and analyzed results aggregating the data in the 46 impacted elements of the four studied systems. Each impacted element included one or more than one LICCI, and we considered that a respondent perceived changes in an impacted element if he/she reported changes in at least one of the LICCI included in that element. To get a deeper understanding of impacts perceived in primary sector activities, we also analyzed impacts in the human system separately, differentiating responses from people with specific professions. Specifically, to analyze perceptions of impacts in agriculture, in this part of the analysis, we only considered the opinion of farmers and agricultural ranchers (*n* = 91), to analyze perceptions of impacts in pastures and livestock we only used the sample of shepherds, ranchers, and agricultural ranchers (*n* = 78), and to analyze perceptions of impacts on bees we only considered the sample of beekeepers (*n* = 31).

The second part of the analysis focused on exploring how individual sociodemographic characteristics and geographic location shape individual perceptions of climate change impacts. We firstly calculated the number of impacts perceived by each informant and compared the average number of LICCI perceived across professions, age ranges, gender, schooling, and Zones. Because the sample did not meet the conditions of normality and homoscedasticity, for our calculations, we used a series of Kruskal–Wallis nonparametric tests.

To assess the degree to which different variables shaped respondent’s reports of climate change impacts, we ran multivariate regressions using our sociodemographic variables (i.e., profession, age, gender, schooling, parents’ and grandparents’ born in Sierra Nevada, years of experience in agropastoral activities, and number of activities conducted in nature) as potential explanatory variables of the total number of impacts perceived by an informant (i.e., *LICCI Index*, our dependent variable). The effect of age was verified by also including the square of the age variable (age^2^), which allows to test for non-linear effects. We used Poisson’s regressions because our dependent variable is discrete with non-negative integer values (Cameron and Trivedi, 1998). To further explore variation, we created four additional variables that, basically, counted the total number of impacts perceived from each system (i.e., *Atmospheric Index*, *Physical Index*, *Biological Index*, and *Human Index*) and ran the same regression model.

We then tested the robustness of our main results by applying some changes to our main model. In our first robustness test, we excluded the 16 women in the sample. In the second robustness model, we excluded the 13 interviewees under 40 years of age, as these participants might have a shorter perspective to compare their observations with previous periods and will rely more on local knowledge transmitted from elder generations. Finally, in our third and fourth robustness tests, instead of the eight Zones, we used the variables “altitude” and “altitude-range” respectively to characterize respondents’ geographical location.

We report $p$-values < 0.05 as indicator of statistical significance. All statistical analyses were carried out with the Stata statistical software program version 13 and the graphical representations of the Microsoft EXCEL program 2016 version.

## Results

We conducted 238 surveys, between 27 and 29 in each Zone, with farmers (*n* = 66), shepherds (*n* = 40), ranchers (*n* = 13), beekeepers (*n* = 31), agricultural ranchers (*n* = 25), and others (*n* = 63). Women composed only 6.7% of the sample, as most women approached refused to participate. Most respondents (76.9%) had between 40 and 69 years of age and only 10.5% had a bachelor or had completed higher studies (Table 1).

**Table 1:** Average number of climate change impacts (LICCI index) reported, by profession, sociodemographic characteristics, and geographical location (Zones)

Variable	Profession Group	Farmers		Shepherds		Ranchers		Beekeepers		Agricultural ranchers		Others		Total sample *(n)* *Average LICCI Index*	
		*n*	*Average LICCI Index*	*n*	*Average LICCI Index*	*n*	*Average LICCI Index*	*n*	*Average LICCI Index*	*n*	*Average LICCI Index*	*n*	*Average LICCI Index*		
Age range	25−39	5	41	1	40	-	-	2	50.5	1	71	4	45	(13)	45.9
	40−54	21	52.3	12	58.2	8	53.9	15	52.9	8	55.7	21	51.7	(85)	53.6
	55−69	20	52.7	22	55.6	5	58.9	12	54.7	9	56.9	30	49	(98)	53.2
	70−84	18	48.3	5	59	-	-	2	58.5	6	53.3	8	45.1	(39)	50.3
	85 and over	2	47.5	-	-	-	-	-	-	1	57	-	-	(3)	50.7
Schooling	No schooling	19	48.5	15	57.9	1		2	62	6	55.2	5	46.8	(48)	53.1
	Primary school completed	25	54.9	17	56.3	8	53.6	9	55.7	18	55.8	25	49.4	(102)	53.9
	Secondary or vocational school completed	15	49.4	8	53.9	3	61.7	15	54.1	1	71	21	48.3	(63)	51.6
	Bachelor or higher school completed.	7	40.9	-		1		5	46.6	-	-	12	51.1	(25)	46.9
Gender	Female	3	46	3	65.3	-	-	5	57.2	1	51	4	42.2	(16)	52.5
	Male	63	50.5	37	55.7	13	55.8	26	53.2	24	56.5	59	49.6	(222)	52.4
Zone	Granada´s surroundings	9	53.1	6	56.8	2	64	6	54	5	60.2	5	42.4	(33)	54.1
	Lanjarón and Lecrín Valley	4	48.5	4	54.2	1	45	6	49.7	4	55	9	47.1	(28)	49.9
	Poqueira Ravine	10	53.2	3	60.7	1	66	3	49	-	-	13	49.3	(30)	52.3
	Trevélez and Bérchules Ravines	12	52.4	1	48	3	61.7	2	71	5	55.8	8	52.1	(31)	54.8
	Ohanes Ravine	5	52.6	8	62.4	1	52	3	60.7	4	57.5	7	51.9	(28)	56.7
	Nacimiento Valley	6	49.5	4	44.5	-	-	5	50.2	5	56	9	58.1	(29)	52.7
	La Calahorra Valley	10	46	6	58.7	1	40	6	54.2	1	63	5	41.8	(29)	50
	Marquesado del Cenete	10	46.9	8	55.1	4	52.2	-	-	1	33	7	44.1	(30)	48.7
Total sample *(n)* *Average LICCI Index*		(66)	50.3	(40)	56.4	(13)	55.8	(31)	53.8	(25)	56.2	(63)	49.2	(238)	52.4

### Perceptions of climate change impacts

**Fig. 2:**
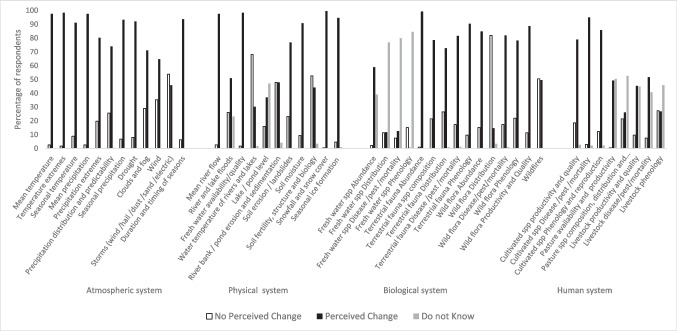
Perception of changes reported by the total sample of respondents on the atmospheric, physical, biological, and human systems

Overall, the number of people who perceived changes in the atmospheric system was larger than the number of people who perceived changes in the rest of the systems (Fig. 2), with more than 90% of respondents perceiving changes in seven out of the 12 impacted elements of the atmospheric system. Changes in five out of the 11 impacted elements of the physical system were perceived by more than 90% of respondents, and changes in nine out of the 15 impacted elements of the biological system were perceived by more than 70% of respondents. In contrast, in the human system, only changes in the three impacted elements of agriculture were perceived by more than 75% of respondents. Overall, the impacted elements for which more respondents perceived changes were *snowfall and snow cover* (perceived by 99.6% of respondents), *terrestrial fauna abundance* (99.2%), *freshwater availability* (98.3%), and *extreme temperatures* (98.3%). Among the ten impacted elements in which changes were most often perceived, four belong to the atmospheric system, four to the physical system, one to the biological, and one to the human system.

Respondents generally agreed about changes in elements of the atmospheric system. Changes in mean and extreme temperatures, changes in mean and seasonal rainfall and droughts, and changes in the duration and timing of seasons were the changes most frequently perceived. Perceptions of changes in elements of the physical system show the greatest disparity of reports. In almost half of the elements of the physical system, changes were perceived by more than 90% of respondents, whereas changes in other elements were less commonly reported (i.e., 68.1% of respondents reported no change in *water temperature of rivers and high mountain lakes*, 52.5% reported no change in *soil fertility, structure, and biology*, and 47.9% reported no change on *riverbank erosion and sedimentation*). Many respondents perceived changes in most of the elements of the biological system, being *changes in abundance of terrestrial fauna* the second element in which changes were most frequently reported (99.2%). Respondents perceived mainly changes in abundance of wild boars (*Sus scrofa*) and mountain goats (*Capra pyrenaica*), but also in bird and insect populations. Many respondents indicated a large increase of starlings (*Sturnus* sp.) in their area and several interviewees claimed that they did not remember having seen them when they were young. A high percentage of respondents (*90.3%*) perceived *changes in terrestrial fauna phenology*, mainly changes in the reproductive and migratory periods of mammals and birds, but also in the activity period of insects, with some shepherds and ranchers reporting that nowadays their livestock has problems with fleas and ticks all year round. A high proportion of respondents (81.5%) reported *changes in terrestrial fauna disease and pests* and shepherds and ranchers commented that transmission of diseases and pests from wild goats and dears is nowadays a persistent problem for livestock in the region. Many respondents also reported changes in elements of wild flora, like *wild flora productivity and quality* (88.7%), noticing a decrease of non-timber forest products like mushrooms and berries, but also changes related to *wild flora abundance* (84.9%) and *wild flora disease/pests* (81.9%). However, more than 75% of informants reported not being aware of changes in fish species, other than a decrease in fish abundance. Some of the oldest interviewees told us that when they were young, people used to “fish while watering the fields,” as trout were so abundant in the river that many got into the irrigation channels and when people opened the floodgates to irrigate the fields, trout ended up in the crops. A few respondents reported changes in fish size, indicating a size increase. “Now we are not allowed to fish trout, so the biggest ones eat most of the fingerlings and the population structure is changing with more adult members and less young fish.”

**Fig. 3:**
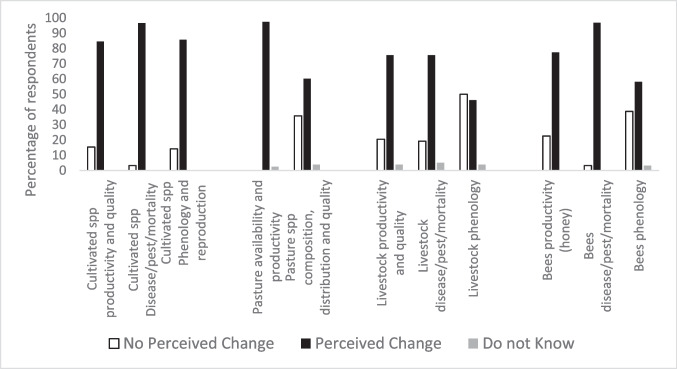
Perception of changes in the human system reported by respondents with professions from the primary sector. Changes in cultivated species (cultivate spp) refer to farmers’ and agricultural ranchers’ reports (*n* = 91); changes in pasture and grassland and changes in livestock refer to shepherds’, ranchers’, and agricultural ranchers’ reports (*n* = 78); and changes in bees refer to beekeepers’ reports (*n* = 31)

Finally, when analyzing the human system, 94.9% of respondents reported *changes in crop pests*, *diseases*, and *mortality* (Fig. 2). The subsample of informants directly dependent on agropastoral professions also reported *changes in the availability of pastures* (97.4%) and *changes in bees’ pest and diseases* (96.8% of beekeepers) (Fig. 3).

### Correlates of climate change impacts reports

On average, each informant reported 52.4 of the 95 local indicators of climate change impacts (LICCI) included in the survey. Shepherds (56.4) and agricultural ranchers (56.2) perceived more impacts (*χ*^2^ = 23.543; *p* = 0.000) than people with other professions. Farmers (50.3) and people with professions outside the primary sector (49.2) perceived the least number of impacts. Groups of respondents in the ranges between 40 and 54 and between 55 and 69 years of age also reported more impacts than people in other age-range groups (*χ*^2^ = 9.54; *p* = 0.049). Men and women reported similar number of LICCI. People with bachelor or higher studies completed perceived less impacts (46.9) than the rest of informants (*χ*^2^ = 10.979; *p* = 0.012). Finally, respondents living in Ohanes Ravine perceived more impacts (56.7) than respondents in other Zones did (*χ*^2^ = 17.762; *p* = 0.013).

We found several associations between sociodemographic characteristics of informants and the number of climate change impacts reported (i.e., LICCI Index) (Table 2). When considering the total number of impacts reported, shepherds and agricultural ranchers reported a statistically significant higher number of impacts than people with other professions (*p* < 0.01; Table 2, Column 1). Age displays an inverted U-shape relation with the number of impacts reported, so although the number of impacts reported by a person increases with his/her age, after a certain point, the relation becomes negative (*p* < 0.01). People with no schooling or who had only completed primary school reported more impacts than people with a bachelor degree or higher studies (*p* < 0.05). We also found that people whose grandparents were born in Sierra Nevada generally reported more climate change impacts than those whose grandparents were born elsewhere, although those whose parents were born in Sierra Nevada reported less impacts than those whose parents were born elsewhere (*p* < 0.05). We found a positive and statistically significant association between the number of impacts reported by a respondent and the number of activities performed in nature by that person (*p* < 0.01). Finally, Zone also showed a statistically significant association with the number of impacts reported, with informants from Granada’s surroundings, Ohanes Ravine, and Trevélez and Bérchules Ravines reporting more impacts than informants from Marquesado del Cenete (our reference category).

**Table 2:** Results of Poisson’s regression showing sociodemographic correlates of climate change impacts reported. Considering the total number of impacts perceived (LICCI Index), and from each of the systems (Atmospheric Index, Physical Index, Biological Index, and Human Index)

	LICCI	Atmospheric	Physical	Biological	Human
	Index	Index	Index	Index	Index
Profession (*others* omitted category)					
Farmers	0.0170	0.0110	0.0585	0.0253	–0.0737
	(0.0267)	(0.0506)	(0.0489)	(0.0498)	(0.0731)
Shepherds	0.1028***	0.0483	0.0810	0.0488	0.3438***
	(0.0317)	(0.0611)	(0.0586)	(0.0596)	(0.0808)
Ranchers	0.0793	0.0475	0.0964	0.0171	0.2257**
	(0.0447)	(0.0858)	(0.0831)	(0.0847)	(0.1112)
Beekeepers	0.0515	–0.0046	0.0188	0.0215	0.2640***
	(0.0328)	(0.0638)	(0.0615)	(0.0612)	(0.0816)
Agricultural ranchers	0.0935***	0.0718	0.0830	0.0279	0.2827***
	(0.0358)	(0.0690)	(0.0661)	(0.0677)	(0.0907)
Age	0.0221***	0.0108	0.0254**	0.0315***	0.0254
	(0.0064)	(0.0119)	(0.0119)	(0.0121)	(0.0170)
Age^2^	–0.0002***	–0.0001	–0.0002**	–0.0003***	–0.0003
	(0.0001)	(0.0001)	(0.0001)	(0.0001)	(0.0001)
Female	–0.0272	0.0440	0.0329	–0.1212	–0.1057
	(0.0383)	(0.0713)	(0.0695)	(0.0744)	(0.1012)
Schooling (*Bachelor or higher school completed* omitted category)					
No schooling	0.0954**	0.0962	0.0995	0.0983	0.0640
	(0.0436)	(0.0834)	(0.0799)	(0.0821)	(0.1142)
Primary school completed	0.0890**	0.1048	0.0751	0.1150	0.0190
	(0.0366)	(0.0702)	(0.0674)	(0.0688)	(0.0955)
Secondary or vocational school completed	0.0671	0.0883	0.0270	0.0929	0.0452
	(0.0363)	(0.0697)	(0.0671)	(0.0681)	(0.0937)
Parents born in Sierra Nevada	–0.1042**	–0.1785	–0.0685	–0.1205	–0.0065
	(0.0514)	(0.0987)	(0.0944)	(0.0959)	(0.1348)
Grandparents born in Sierra Nevada	0.0509**	0.0968**	0.0347	0.0425	0.0172
	(0.0246)	(0.0478)	(0.0450)	(0.0460)	(0.0630)
Years of experience agropastoral activities	–0.0016	–0.0019	–0.0012	–0.0019	–0.0009
	(0.0011)	(0.0020)	(0.0020)	(0.0021)	(0.0031)
Number of activities in nature	0.0422***	0.0210	0.0201	0.0531***	0.1025***
	(0.0099)	(0.0190)	(0.0185)	(0.0186)	(0.0256)
Zone (*Marquesado del Cenete* omitted category)					
Zone Granada´s surroundings	0.0777**	–0.1057	0.0711	0.2602***	0.1060
	(0.0378)	(0.0711)	(0.0697)	(0.0717)	(0.1003)
Zone Lanjarón and Lecrín Valley	0.0034	–0.1551**	0.0167	0.0942	0.1159
	(0.0406)	(0.0769)	(0.0749)	(0.0777)	(0.1054)
Zone Poqueira Ravine	0.0505	–0.0420	0.0310	0.1786**	0.0437
	(0.0390)	(0.0719)	(0.0723)	(0.0741)	(0.1063)
Zone Trevélez and Bérchules Ravines	0.1009***	–0.0258	0.0666	0.1770**	0.2818***
	(0.0386)	(0.0717)	(0.0714)	(0.0743)	(0.1012)
Zone Ohanes Ravine	0.1071***	–0.0126	0.1491**	0.1998***	0.1018
	(0.0390)	(0.0723)	(0.0710)	(0.0753)	(0.1049)
Zone Nacimiento Valley	0.0438	–0.1345	0.1015	0.1140	0.1490
	(0.0397)	(0.0749)	(0.0723)	(0.0765)	(0.1043)
Zone La Calahorra Valley	0.0146	–0.0490	0.0535	0.0400	0.0351
	(0.0392)	(0.0718)	(0.0715)	(0.0764)	(0.1053)
_cons	3.0705***	2.2165***	1.7604***	1.5044***	0.8775
	(0.1918)	(0.3596)	(0.3589)	(0.3627)	(0.5009)
*N*	238	238	238	238	238

Standard errors in parentheses ** *p* < 0.05, *** *p* < 0.01

We conducted a similar analysis using as dependent variable the total number of impacts perceived in each of the systems analyzed. The number of impacts reported on elements of the atmospheric system (i.e., *Atmospheric Index*) was only associated with the variables that capture whether the informant’s grandparents were born in Sierra Nevada. We also found that people from Lanjarón and Lecrín Valley reported fewer impacts in the atmospheric system than people from other Zones (Column 2, Table 2). The number of impacts on elements of the physical system (i.e., *Physical Index*) only showed a statistically significant and positive association with living in Ohanes Ravine (Column 3, Table 2), where an informant told to us: “when I was a child, the spring of my village had a flow of ‘two bodies’ [a local measure of volume] and now there is less than ‘one arm.” Age of respondents, number of activities performed in nature, and Zone (i.e., living in Granada’s surroundings, Poqueira Ravine, Trevélez and Bérchules Ravines, and Ohanes Ravine) bear a positive association with the number of impacts perceived in the biological system (i.e., *Biological Index*, Column 4 in Table 2). Finally, respondent’s profession and total number of activities performed in nature were associated in a statistically significant way with the number of climate change impacts perceived in the human system (i.e., *Human Index*). Particularly, except *farmers*, people with all other professions perceived more impacts than respondents in the group of *others* (i.e., professions not related to the primary sector) (Column 5, Table 2). People living in Trevélez and Bérchules Ravines also perceived more impacts in the human system than people living in other Zones.

Results from our robustness test only minimally deviate from our main results (Online Resource 2). Thus, in the regression using only the subsample of men (*n* = 222), the variable that captures whether parents were born in Sierra Nevada becomes statistically insignificant and the category that captures respondents secondary or vocational studies becomes significant. All other statistically significant associations found in the main model remain. In the regression excluding people under 40 years of age (*n* = 225), only the age and the variables that capture whether the informant had parents and grandparents born in Sierra Nevada become statistically insignificant. Finally, in the two models in which the variable that capture the Zone where a resident lives is superseded by the variable altitude and the altitude-range respectively, we found that neither of these two variables bears a statistically significant association with the LICCI Index.

## Discussion

Findings from this work show that Sierra Nevada inhabitants perceive multiple local indicators of climate change impacts. Changes in snow and hydric resources, abundance of terrestrial fauna, and extreme temperatures were reported by more than 98% of respondents. However, when looking at the full range of impacts in the area, we also found that the number and type of impacts reported are shaped by respondents’ sociodemographic characteristics and geographical location.

Before discussing the importance of these results, we present some sampling and measurement biases that call for caution in interpreting them. First, we acknowledge that our sample might be biased. Although we aimed for a balanced sample, due to particularities of the study region, this was not feasible. Thus, we could only interview a low number of people over 75 years, as many people in this age range preferred not to do the survey and delegated it in a younger family member. Additionally, local cultural norms resulted in higher rates of refusal to participate among women than among men. Finally, orographic and environmental differences produce an unbalanced geographical concentration of some professions, which is reflected in the population distribution across activities and Zones in our sample. We have tried to assess the effect of these sampling biased in our results by testing our model with different subsamples. Second, our results might also be affected by measurement errors resulting from informants providing—voluntarily or involuntarily—inaccurate information, as it often happens when referring to issues occurring in past times (Armah et al. 2015). We cannot correct for this potential bias, which might affect our results in unknown magnitude and direction. Finally, an important caveat to keep in mind when interpreting our results relates to the influence of other drivers of change. As elsewhere, in our study region, several other drivers of change interact with climate change generating environmental impacts (Junqueira et al. 2021; Pörtner et al. 2021). In our survey, we asked about environmental changes potentially driven by climate change, but as we did not ask particularly about other drivers of change, the role of climate change as driver might be overestimated.

A main finding of this work is that, drawing on local knowledge, inhabitants of Sierra Nevada are aware of multiple climate change impacts, particularly changes in the atmospheric systems, with changes in snowfall and snow cover, changes in freshwater availability, and changes in terrestrial fauna abundance being the three impacted elements most often reported. Overall, the finding concords with previous literature reviews of local observations of climate change impacts (Savo et al. 2016; Reyes-García et al. 2019) and recent research in the area (Garteizgogeascoa et al. 2020) signaling that local inhabitants perceive changes attributable to climate change impacts. Changes directly related to the water-cycle (e.g., changes in rainfall, changes in freshwater availability, changes in ice and snow) were reported by most informants, as it is also the case in other mountain regions (Xu et al. 2009; Postigo 2014; Shijin and Dahe 2015; Córdova et al. 2019; Huang et al. 2019). Indeed, Sierra Nevada has experienced a decline in water resources during the last century (Jódar et al. 2017), including changes in rainfall trends, with less rainy summers (Ruiz Sinoga et al. 2011), dryer and longer autumns (Machado et al. 2011), and shorter snow periods than 50 years ago (Bonet et al. 2016). Data series collected since 1960 showed a significant negative trend in rainfall in 43% of the Sierra Nevada territory (Pérez-Luque et al. 2016).

It is also interesting to highlight the high percentage of respondents who reported impacts on elements of the biological system, an essential information for bridging scientific and local knowledge systems according to a recent survey to climate change experts (García-del-Amo et al. 2020). Changes in abundance of terrestrial wild fauna, e.g., Iberian ibex (*Capra pyrenaica*), wild boar (*Sus scrofa*), or starling (*Sturnus* sp.), were the changes most frequently reported. While some of these reports dovetail with information from instrumental measurements (e.g., the increase of Iberian ibex population (Granados et al. 2020)), others are new to the scientific literature in the area. This is the case, for example, of reports on the increase of the population of starlings, to the detriment of other local species, a change that has been reported in other parts of the world (e.g., Craig 2020), but not locally. Changes in terrestrial fauna also include observations in changes in behavior, distribution, and phenology of reptiles and insects, matching findings from other studies in mountain areas (Postigo 2014; Ingty 2017; Lamsal et al. 2017). For example, shepherds reported phenological changes in the fleas, ticks, horseflies, wasps, and bees, as it has also been reported by researchers from the Global Observatory of Sierra Nevada (Hódar and Zamora 2004; Illán et al. 2012; Menéndez et al. 2014). Similarly, informants’ reports on changes in wild flora abundance and productivity and wild flora diseases match with the increase of pine processionary moths during the last 60 years, which is also reported in the literature (Ros-Candeira et al. 2019). Differently, informants reported few impacts in freshwater fish and the reported impacts in abundance and size differ from results of recent studies in the area (Galiana-García et al. 2016), a finding that we attribute to the effects that the fishing prohibition implemented with the declaration of the national park (1999) in eroding local practices and knowledge system (Fox et al. 2009; Santos and Sampaio 2013). Informants also perceived changes in the human system, being changes in crop productivity and quality, crop disease and pests, and crops phenology the changes most reported by informants. Importantly and as in other mountain pastoral communities (Wang and Cao 2015; Tilahun et al. 2017; Uprety et al. 2017; Kharumnuid et al. 2018; Córdova et al. 2019), Sierra Nevada respondents with professions directly devoted to the primary sector also perceived several changes in availability and quality of pastures and pest and diseases in livestock species and bees. To us, the richness of reports of change documented through local knowledge could be at the basis for efforts to explore synergies between local ecological knowledge and science. In this sense, researchers recognized that it could improve our understanding of climate change impacts at a local scale mainly in the biological and human system (see García-del-Amo et al. 2020).

The second important finding of this work is that reports of climate change impacts are patterned by respondents’ sociodemographic characteristics and geographic location. Regarding sociodemographic characteristics, we found that variables capturing respondents’ bonding with the territory (i.e., number of activities performed in nature, grandparents being born in Sierra Nevada, and age) showed a positive association with the number of climate change impacts reported by the person, whereas having high levels of formal education showed a negative association with the number of climate change impacts reported by the person, probably because formal education often proxies for local knowledge erosion (Iselin 2011; Benyei et al. 2020). Independently of their profession, the number of activities that an informant has performed in nature was directly related with the number of impacts reported. Moreover, in consonance with this finding, we also found that shepherds and agricultural ranchers reported more impacts than people with other professions, probably because their activities demand long periods of time in nature alone, which facilitates observing and understanding the environment. Indeed, the relevance and accuracy of shepherd’s and herder communities’ knowledge have also been found among Spanish transhumant shepherds (Oteros-Rozas et al. 2013), nomadic herders in Mongolia (Fassnacht et al. 2018), or Sami herders in Finland (Riseth et al. 2011). The importance of bonding with the territory in shaping respondents’ reports of climate change impacts was also reflected in the association with the variable that captured whether the informant had grandparents born in Sierra Nevada and informant’s age. Interestingly, we also found a negative association between the number of impacts reported and having parents been born in the area. To us, these apparently contradictory findings might reflect differences in knowledge among respondents from families migrating from other regions (Brandt et al. 2013; Ma et al. 2019) compared with people from families with more than a century of permanence in Sierra Nevada. Respondents under 40 years perceived fewer impacts than other groups due to their experiential range being smaller to compare changes happening in the last 25 years with previous times. However, an agricultural rancher from this group reported the third-highest number of impacts, which might indicate the influence of local knowledge transmitted by elder generations. It is also important to highlight that age does not show a linear association with the number of climate change impacts reported, as the eldest respondents reported fewer impacts than younger respondents. Although the finding could just reflect cognitive degeneration (Bermejo-Pareja et al. 2008), we argue that it could also reflect that old informants are less active professionally, for which they might not be updating their knowledge of subtle changes as frequently as those who continue working actively (Oteros-Rozas et al. 2013; Klein et al. 2014).

In addition to individual sociodemographic characteristics, respondent’s geographic location also shaped the number and type of climate change impacts reported, which might suggest that climate change impacts vary event at small geographical scales. For example, people in Ohanes Ravine reported more impacts in the physical system than people from other Zones. The Ohanes Ravine is located in the south-east region, far from the last snowfields of the summer and with highest hydric stress (Raso Nadal 2011), which directly affects rivers’ flow and duration, soil moisture, and freshwater availability in this Zone. Similarly, respondents in Trevélez and Bérchules Ravines, who proportionally have a higher dependence on agriculture than respondents in other Zones, reported more impacts in elements of the human system. In this Zone, located in the windward side of Sierra Nevada, villages are at high elevation and close to the snowfields that remain the longest at summer, with secured access to water. As informants reported, temperature increase has allowed them to lengthen their agricultural production season by almost 2 months, although the temperature increase has also favored the proliferation of pests, as has been reported in other mountain areas (Taboada et al. 2017; Meldrum et al. 2018). It is worth mentioning, however, that our robustness test shows that village altitude and altitude-range of the different Zones are not significantly associated with the number of impacts reported, probably because altitude is only one among multiple factors. For example, people in La Calahorra Valley, located at a high altitude but in the leeward side of the mountain, have not been able to maintain such a productive agricultural system as people in Bérchules and Trevélez Ravines due to the scarcity of hydric resources, which is influenced by the Foehn and the NAO effects (Herrero and Polo 2016; Pérez-Luque et al. 2016). Similarly, people in Lanjarón and Lecrín valley, located at 772 MSL, reported less impacts in the atmospheric system than people in other Zones located at higher altitudes, probably due to the local effect of a big dam built in this Zone 16 years ago, which acts as a buffer decreasing high summer temperatures, generating a microclimate (Miller 2005; Degu et al. 2011).

Geographic location also shapes reports of changes in elements of the biological system, which are attributed to multiple drivers of change. For example, changes in the abundance of wildlife were more often perceived in Granada surroundings, Poqueira Ravine, Bérchules and Trevélez Ravines, and Ohanes Ravine. Respondents in these communities argued that damages in crops and orchards have increased due to the presence of wild boars and goats who now lack food in the mountain because of decreased rainfall and land use change generated by pine reforestation (Zamora et al. 2016). However, in Granada surroundings and Poqueira Ravine, Zones with the highest touristic pressure of Sierra Nevada, respondents recognized that wild fauna is also losing its habitat due to the pressure of the touristic sector, which might result in an increase of their presence in lower areas. In Trevélez and Bérchules Ravines, people claimed that wild animals are moving from other Zones because they still have a productive agriculture, which attract starving animals displaced from other areas. On the contrary, respondents from Ohanes Ravine attribute the increase in the abundance of animals and their impact on crops to the greater hydric stress in that Zone compared to other areas in Sierra Nevada, to the lower hunting control, and to the displacement of groups of deer from Filabres, the mountain range parallel to the Sierra Nevada.

All in all, these results suggest that climate change impacts can be different even at small geographical scales, as it has also been showed in other European forests (Mina et al. 2017). Moreover, our results add to this previous finding suggesting that differentiated impacts at small geographical area might be larger when climate change impacts interact with other drivers of environmental change. The implication of this finding is that more locally grounded data is needed to develop accurate climate change models and adaptation measures (Xu et al. 2009; Cramer et al. 2014; Ford et al. 2016; Reyes-García et al. 2019).

## Conclusion

Local inhabitants of Sierra Nevada report multiple climate change impacts on their local environment and livelihoods. Many of these reports match with results obtained by scientists, although not all reports from local knowledge have comparable instrumental data, which highlights the potential of exploring synergies between different knowledge systems to improve our understanding of climate change impacts. Although respondents frequently reported changes in elements of the atmospheric system, they also highlighted changes in hydric resources and abundance of terrestrial fauna directly impacting their traditional livelihoods. Local adaptation policies should focus on minimizing the impacts of such changes. In the study area, these could be done, for example, through the conservation of traditional irrigation systems and the social structure that supports them. Finally, an important finding of our work is that local reports of climate change impacts are shaped by informants’ sociodemographic characteristics (i.e., level of bonding to the territory, profession, formal education, grandparents’ being born in Sierra Nevada) and their geographic location. Beyond the specific characteristics that shape intracultural knowledge distribution, the finding that reports of climate change are socially patterned warns for caution when referring to local knowledge systems, as there might be important variations within individuals in a same community. The finding also highlights the need to engage a diversity of local populations in developing local policies for co-management and climate change adaptation. Similarly, the finding that climate change impacts can be different even at small geographic scales should be considered when developing adaptation policies, recognizing the diversity of local realities of affected communities.

## Supplementary Information

Below is the link to the electronic supplementary material.

Supplementary file1 (DOCX 79 KB)

Supplementary file2 (DOCX 22 KB)
