# Supplementary material for: Local reports of climate change impacts in Sierra Nevada, Spain: sociodemographic and geographical patterns
Source: Reg Environ Change. 2022 Dec 16;23(1):14. doi: 10.1007/s10113-022-01981-5 (PMC9758096; doi:10.1007/s10113-022-01981-5)
Supplement: Supplementary file 1 — Supplementary file1 (DOCX 79 KB) [file 10113_2022_1981_MOESM1_ESM.docx]

**Local reports of climate change impacts in Sierra Nevada, Spain: Sociodemographic and geographical patterns**

**Regional Environmental Change**

David García-del-Amo^*^; Peter Graham Mortyn; Victoria Reyes-García

* David.Garcia.delAmo@uab.cat

Institut de Ciència I Tecnologia Ambientals, Universitat Autònoma de Barcelona, Columnes S/N. Building ICTA-IPC(Z) UAB Campus, 08193, Bellaterra - Barcelona, Spain

Department of Geography, Universitat Autònoma de Barcelona, 08193, Bellaterra - Barcelona, Spain,

Online Resource 1

| Subsystem | Impacted Element | LICCI | **No Change** | | **Change** | | **Don´t Know** | | **Zone** | | **Livelihood** | | |  |
| --- | --- | --- | --- | --- | --- | --- | --- | --- | --- | --- | --- | --- | --- | --- |
|  |  |  | **n** | **%** | **n** | **%** | **n** | **%** | **Χ^2^** | **p** | **Χ^2^** | **p** | |  |
| Atmospheric system | | | | | | | | | | | | |  |  |
| **TEMPERATURE** | Mean temperature | Change mean temperature | 10 | 4.20 | 228 | 95.80 | 0 | 0.00 | 10.1002 | .1830 | 3.2625 | 0.6596 | |  |
|  |  | Change frequency of unusual temperature | 56 | 23.53 | 182 | 76.47 | 0 | 0.00 | 24.3031 | 0.0010* | 3.2045 | 0.6685 | |  |
|  | *Mean temperature* | | **6** | **2.52** | **232** | **97.48** | **0** | **0.00** | **29.8049** | **0.0001*** | **3.3723** | **0.6428** | |  |
|  | Extreme temperature | Change number of extremely cold days | 14 | 5.88 | 224 | 94.12 | 0 | 0.00 | 4.5128 | 0.7192 | 4.5515 | 0.4730 | |  |
|  |  | Change number of extremely hot days | 35 | 14.71 | 203 | 85.29 | 0 | 0.00 | 3.2045 | 0.8655 | 5.5706 | 0.3503 | |  |
|  |  | Change frequency of heat waves | 45 | 18.91 | 193 | 81.09 | 0 | 0.00 | 18.6091 | 0.0095* | 3.3857 | 0.6407 | |  |
|  | *Extreme Temperature* | | **4** | **1.68** | **234** | **98.32** | **0** | **0.00** | **6.9893** | **0.4300** | **2.2399** | **0.8151** | |  |
|  | Seasonal temperature | Change frequency of heat or cold periods discordant with the time of the year | 21 | 8.82 | 217 | 91.18 | 0 | 0.00 | 9.0848 | 0.2466 | 5.0572 | 0.4089 | |  |
|  | *Seasonal temperature* | | **21** | **8.82** | **217** | **91.18** | **0** | **0.00** | **9.0848** | **0.2466** | **5.0572** | **0.4089** | |  |
| **PRECIPITATION** | Mean precipitation | Changes number of rainy days | 6 | 2.52 | 232 | 97.48 | 0 | 0.00 | 4.2382 | 0.7520 | 4.1279 | 0.5311 | |  |
|  | *Mean precipitation* | | **6** | **2.52** | **232** | **97.48** | **0** | **0.00** | **4.2382** | **0.7520** | **4.1279** | **0.5311** | |  |
|  | Extreme precipitation | Change number of extreme rainfall days | 47 | 19.75 | 191 | 80.25 | 0 | 0.00 | 25.3627 | 0.0007* | 4.6480 | 0.4603 | |  |
|  | *Extreme precipitation* | | **47** | **19.75** | **191** | **80.25** | **0** | **0.00** | **25.3627** | **0.0007*** | **4.6480** | **0.4603** | |  |
|  | Precipitation distribution, variability, and predictability | Change frequency of unpredictable rainfall | 61 | 25.63 | 176 | 73.95 | 1 | 0.42 | 11.4695 | 0.1194 | 2.8377 | 0.7250 | |  |
|  | *Precipitation distribution, variability, and predictability* | | **61** | **25.63** | **176** | **73.95** | **1** | **0.42** | **11.4695** | **0.1194** | **2.8377** | **0.7250** | |  |
|  | Seasonal precipitation | Change the amount of rainfall per season | 16 | 6.72 | 222 | 93.28 | 0 | 0.00 | 15.8092 | 0.0269* | 5.6189 | 0.3451 | |  |
|  | *Seasonal precipitation* | | **16** | **6.72** | **222** | **93.28** | **0** | **0.00** | **15.8092** | **0.0269*** | **5.6189** | **0.3451** | |  |
|  | Drought | Change frequency of drought periods | 26 | 10.92 | 212 | 89.08 | 0 | 0.00 | 9.3845 | 0.2262 | 10.0149 | 0.0748 | |  |
|  |  | Change the length of drought periods | 21 | 8.82 | 217 | 91.18 | 0 | 0.00 | 7.5956 | 0.3696 | 3.7058 | 0.5925 | |  |
|  | *Drought* | | **19** | **7.98** | **219** | **92.01** | **0** | **0.00** | **10.5855** | **0.1577** | **9.2264** | **0.1004** | |  |
|  | Clouds and fog | Change number of fogy days | 69 | 28.99 | 169 | 71.01 | 0 | 0.00 | 20.2657 | 0.0050* | 12.5265 | 0.0282* | |  |
|  | *Clouds and fog* | | **69** | **28.99** | **169** | **71.01** | **0** | **0.00** | **20.2657** | **0.0050*** | **12.5265** | **0.0282*** | |  |
| **AIR MASSES** | Wind | Change wind speed | 143 | 60.08 | 95 | 39.92 | 0 | 0.00 | 21.9817 | 0.0026* | 3.5186 | 0.6206 | |  |
|  |  | Change number of windy days | 97 | 40.76 | 141 | 59.24 | 0 | 0.00 | 15.7573 | 0.0274* | 1.4550 | 0.9182 | |  |
|  | *Wind* | | **84** | **35.29** | **154** | **64.70** | **0** | **0.00** | **22.7456** | **0.0019*** | **2.2638** | **0.8116** | |  |
|  | Storms | Change number of sand or dust storms | 128 | 53.78 | 109 | 45.80 | 1 | 0.42 | 39.7195 | 0.0000* | 9.1349 | 0.1038 | |  |
|  | *Storms* | | **128** | **53.78** | **109** | **45.80** | **1** | **0.42** | **39.7195** | **0.0000*** | **9.1349** | **0.1038** | |  |
| **SEASONS** | Duration and timing of seasons | Change the length of seasons | 22 | 9.24 | 216 | 90.76 | 0 | 0.00 | 9.1047 | 0.2452 | 1.8849 | 0.8648 | |  |
|  |  | Change transition speed between seasons | 72 | 30.25 | 166 | 69.75 | 0 | 0.00 | 31.0251 | 0.0001* | 2.0030 | 0.8487 | |  |
|  | *Duration and timing of seasons* | | **15** | **6.30** | **223** | **93.70** | **0** | **0.00** | **23.0977** | **0.0016*** | **0.5511** | **0.9901** | |  |
| Physical system | | | | | | | | | | | | | | |
| **FRESHWATER** | Mean river flow | Change in mean river flow | 12 | 5.04 | 226 | 94.96 | 0 | 0.00 | 13.4574 | 0.0617 | 14.8474 | 0.0110* | |  |
|  |  | Change high mountain springs flow | 10 | 4.20 | 208 | 87.39 | 20 | 8.40 | 18.1992 | 0.0111* | 4.2261 | 0.5173 | |  |
|  | *Mean river flow* | | **6** | **2.52** | **232** | **97.48** | **0** | **0** | **16.3390** | **0.0220*** | **6.8599** | **0.2313** | |  |
|  | River and lake floods | Change surface flooded by floods | 73 | 30.67 | 109 | 45.80 | 56 | 23.53 | 65.5495 | 0.0000* | 16.8785 | 0.0047* | |  |
|  |  | Change number of floods and overflows of rivers | 62 | 26.05 | 121 | 50.84 | 55 | 23.11 | 55.7326 | 0.0000* | 11.8848 | 0.0364* | |  |
|  | *River and lake floods* | | **62** | **26.05** | **121** | **50.84** | **55** | **23.11** | **63.2240** | **0.0000*** | **14.8373** | **0.0111*** | |  |
|  | Fresh water availability/quality | Change duration of temporal rivers | 24 | 10.08 | 214 | 89.92 | 0 | 0.00 | 15.6958 | 0.0280* | 6.5482 | 0.2565 | |  |
|  |  | Change duration of irrigation water | 20 | 8.40 | 214 | 89.92 | 4 | 1.68 | 4.6263 | 0.7055 | 7.3595 | 0.1952 | |  |
|  |  | Change duration of high mountain springs | 15 | 6.30 | 202 | 84.87 | 21 | 8.82 | 20.0756 | 0.0054* | 4.6444 | 0.4608 | |  |
|  | *Fresh water availability/quality* | | **4** | **1.68** | **234** | **98.32** | **0** | **0** | **8.6850** | **0.2760** | **3.0603** | **0.6907** | |  |
|  | Water temperature of rivers and lakes | Change water temperature of rivers and lakes | 162 | 68.07 | 72 | 30.25 | 4 | 1.68 | 10.3360 | 0.1703 | 3.1980 | 0.6695 | |  |
|  | *Water temperature of rivers and lakes* | | **162** | **68.07** | **72** | **30.25** | **4** | **1.68** | **10.3360** | **0.1703** | **3.1980** | **0.6695** | |  |
|  | Lake level | Change duration of high mountain lakes | 38 | 15.97 | 88 | 36.97 | 112 | 47.06 | 20.3741 | 0.0048* | 1.9723 | 0.8530 | |  |
|  | *Lake level* | | **38** | **15.97** | **88** | **36.97** | **112** | **47.06** | **20.3741** | **0.0048*** | **1.9723** | **0.8530** | |  |
|  | Riverbank erosion and sedimentation | Change in rivers´ erosion grade | 114 | 47.90 | 114 | 47.90 | 10 | 4.20 | 13.3446 | 0.0641 | 3.6464 | 0.6014 | |  |
|  | *Riverbank erosion and sedimentation* | | **114** | **47.90** | **114** | **47.90** | **10** | **4.20** | **13.3446** | **0.0641** | **3.6464** | **0.6014** | |  |
| **SOIL & LAND** | Soil erosion/ landslides | Change soil erosion | 100 | 42.02 | 134 | 56.30 | 4 | 1.68 | 6.1630 | 0.5209 | 3.6861 | 0.5954 | |  |
|  |  | Change number of landslides | 112 | 47.06 | 126 | 52.94 | 0 | 0.00 | 35.9058 | 0.0000* | 1.6627 | 0.8936 | |  |
|  | *Soil erosion/ landslides* | | **55** | **23.11** | **183** | **76.89** | **0** | **0** | **17.6480** | **0.0140*** | **1.9900** | **0.8505** | |  |
|  | Soil moisture | Change soil humidity | 22 | 9.24 | 216 | 90.76 | 0 | 0.00 | 10.1386 | 0.1809 | 3.7439 | 0.5868 | |  |
|  | *Soil moisture* | | **22** | **9.24** | **216** | **90.76** | **0** | **0.00** | **10.1386** | **0.1809** | **3.7439** | **0.5868** | |  |
|  | Soil fertility, structure, and biology | Change percentage of organic matter in the soil | 125 | 52.52 | 105 | 44.12 | 8 | 3.36 | 26.6071 | 0.0004* | 13.1292 | 0.0222* | |  |
|  | *Soil fertility, structure, and biology* | | **125** | **52.52** | **105** | **44.12** | **8** | **3.36** | **26.6071** | **0.0004*** | **13.1292** | **0.0222*** | |  |
| **ICE & SNOW** | Snowfall and snow cover | Change amount of snowfall per year | 2 | 0.84 | 236 | 99.16 | 0 | 0.00 | 5.8809 | 0.5537 | 2.8760 | 0.7191 | |  |
|  |  | Change frequency of snowfalls after winter | 48 | 20.17 | 190 | 79.83 | 0 | 0.00 | 41.5131 | 0.0000* | 12.7043 | 0.0263* | |  |
|  |  | Change duration of snowfields | 5 | 2.10 | 232 | 97.48 | 1 | 0.42 | 4.7433 | 0.6913 | 5.3863 | 0.3706 | |  |
|  |  | Change snow cover extension | 10 | 4.20 | 227 | 95.38 | 1 | 0.42 | 5.3879 | 0.6127 | 7.0467 | 0.2172 | |  |
|  |  | Change size of snowfields | 26 | 10.92 | 209 | 87.82 | 3 | 1.26 | 12.0057 | 0.1004 | 3.4054 | 0.6378 | |  |
|  | *Snowfall and snow cover* | | **1** | **0.42** | **237** | **99.58** | **0** | **0** | **34.8670** | **0.0000*** | **10.0400** | **0.0741** | |  |
|  | Seasonal ice formation | Change presence of ice sheet on rivers and lakes | 37 | 15.55 | 198 | 83.19 | 3 | 1.26 | 13.1739 | 0.0680 | 6.4293 | 0.2667 | |  |
|  |  | Change Ice sheet thickness | 14 | 5.88 | 221 | 92.86 | 3 | 1.26 | 7.1580 | 0.4130 | 10.5494 | 0.0611 | |  |
|  | *Seasonal ice formation* | | **11** | **4.62** | **225** | **94.54** | **2** | **0.84** | **11.3520** | **0.1240** | **7.8775** | **0.1631** | |  |
| Biological system | | | | | | | | | | | | | |  |
| **FRESHWATER WILD FAUNA** | Freshwater Fish spp Abundance | Change abundance of Fish | 5 | 2.10 | 140 | 58.82 | 93 | 39.08 | 59.6577 | 0.0000* | 6.9095 | 0.2275 | |  |
|  | *Freshwater Fish spp Abundance* | | **5** | **2.10** | **140** | **58.82** | **93** | **39.08** | **59.6577** | **0.0000*** | **6.9095** | **0.2275** | |  |
|  | Freshwater Fish spp Distribution and migration | Change altitudinal distribution of Fish | 27 | 11.34 | 28 | 11.76 | 183 | 76.89 | 31.6772 | 0.0000* | 9.7433 | 0.0828 | |  |
|  | *Freshwater Fish spp Distribution and migration* | | **27** | **11.34** | **28** | **11.76** | **183** | **76.89** | **31.6772** | **0.0000*** | **9.7433** | **0.0828** | |  |
|  | Freshwater Fish spp Disease/ pest/ mortality | Change frequency of diseases/parasites in Fish | 43 | 18.07 | 2 | 0.84 | 193 | 81.09 | 5.4678 | 0.6031 | 4.5824 | 0.4689 | |  |
|  |  | Change frequency of mutations/deformities in Fish | 45 | 18.91 | 0 | 0.00 | 193 | 81.09 | 0.0000 | 1.0000 | 0.0000 | 1.0000 | |  |
|  |  | Change size of Fish | 18 | 7.56 | 30 | 12.61 | 190 | 79.83 | 51.0591 | 0.0000* | 2.8958 | 0.7160 | |  |
|  | *Freshwater Fish spp Disease/ pest/ mortality* | | **18** | **7.56** | **30** | **12.60** | **190** | **79.83** | **50.8982** | **0.0000*** | **2.9153** | **0.7130** | |  |
|  | Freshwater Fish spp Phenology | Change reproduction dates of Fish | 36 | 15.13 | 1 | 0.42 | 201 | 84.45 | 6.9333 | 0.4359 | 2.6061 | 0.7604 | |  |
|  | *Freshwater Fish spp Phenology* | | **36** | **15.13** | **1** | **0.42** | **201** | **84.45** | **6.9333** | **0.4359** | **2.6061** | **0.7604** | |  |
| **TERRESTRIAL WILD FAUNA** | Terrestrial Wild fauna Abundance | Change abundance Terrestrial animals (mammals, reptiles | 9 | 3.78 | 228 | 95.80 | 1 | 0.42 | 11.0819 | 0.1351 | 7.4738 | 0.1877 | |  |
|  |  | Change abundance Birds | 10 | 4.20 | 227 | 95.38 | 1 | 0.42 | 9.8269 | 0.1986 | 10.3742 | 0.0653 | |  |
|  |  | Change abundance Insects | 34 | 14.29 | 204 | 85.71 | 0 | 0.00 | 11.0380 | 0.1370 | 2.1492 | 0.8281 | |  |
|  |  | Change abundance Wild Hives | 8 | 3.36 | 216 | 90.76 | 14 | 5.88 | 15.0140 | 0.0358* | 4.8258 | 0.4375 | |  |
|  | *Terrestrial Wild fauna Abundance* | | **2** | **0.84** | **236** | **99.16** | **0** | **0** | **9.3493** | **0.2285** | **6.2425** | **0.2833** | |  |
|  | Terrestrial Wild fauna composition (assemblage of species) | Change presence new Bird | 56 | 23.53 | 179 | 75.21 | 3 | 1.26 | 6.5485 | 0.4773 | 4.0285 | 0.5453 | |  |
|  |  | Change presence new Insects | 191 | 80.25 | 33 | 13.87 | 14 | 5.88 | 33.3459 | 0.0000* | 5.2387 | 0.3874 | |  |
|  | *Terrestrial Wild fauna composition* | | **51** | **21.43** | **187** | **78.57** | **0** | **0** | **13.6719** | **0.0573** | **3.3810** | **0.6415** | |  |
|  | Terrestrial Wild fauna Distribution and migration | Change altitudinal distribution Terrestrial animals | 68 | 28.57 | 160 | 67.23 | 10 | 4.20 | 42.5654 | 0.0000* | 2.4553 | 0.7832 | |  |
|  |  | Change altitudinal distribution Birds | 203 | 85.29 | 31 | 13.03 | 4 | 1.68 | 38.0769 | 0.0000* | 7.8184 | 0.1665 | |  |
|  |  | Change altitudinal distribution Insects | 213 | 89.50 | 22 | 9.24 | 3 | 1.26 | 13.4623 | 0.0616 | 7.2349 | 0.2038 | |  |
|  | *Terrestrial Wild fauna Distribution and migration* | | **63** | **26.47** | **173** | **72.69** | **2** | **0.84** | **44.8488** | **0.0000*** | **7.4719** | **0.1878** | |  |
|  | Terrestrial Wild fauna Disease/ pest/ mortality | Change abundance of diseases/parasites in Terrestrial animals | 50 | 21.01 | 184 | 77.31 | 4 | 1.68 | 13.1548 | 0.0684 | 9.8153 | 0.0806 | |  |
|  |  | Change frequency of mutations/deformities in Terrestrial animals | 230 | 96.64 | 5 | 2.10 | 3 | 1.26 | 12.3099 | 0.0908 | 3.9033 | 0.5634 | |  |
|  |  | Change abundance of starving Terrestrial animals | 134 | 56.30 | 99 | 41.60 | 5 | 2.10 | 27.1373 | 0.0003* | 9.2643 | 0.0990 | |  |
|  |  | Change abundance of diseases/parasites in Birds | 227 | 95.38 | 7 | 2.94 | 4 | 1.68 | 14.4338 | 0.0440* | 6.7070 | 0.2434 | |  |
|  |  | Change frequency of mutations/deformities in Birds | 233 | 97.90 | 1 | 0.42 | 4 | 1.68 | 6.9333 | 0.4359 | 2.7778 | 0.7342 | |  |
|  |  | Change size of Birds | 207 | 86.97 | 28 | 11.76 | 3 | 1.26 | 20.1350 | 0.0053* | 6.8342 | 0.2333 | |  |
|  | *Terrestrial Wild fauna Disease/ pest/ mortality* | | **41** | **17.23** | **194** | **81.51** | **3** | **1.26** | **34.9853** | **0.0000*** | **7.7262** | **0.1720** | |  |
|  | Terrestrial Wild fauna Phenology | Change hibernation dates Terrestrial animals | 112 | 47.06 | 121 | 50.84 | 5 | 2.10 | 14.4164 | 0.0443* | 8.7656 | 0.1188 | |  |
|  |  | Change reproduction dates Terrestrial animals | 170 | 71.43 | 60 | 25.21 | 8 | 3.36 | 7.4480 | 0.3838 | 11.4410 | 0.0433* | |  |
|  |  | Change migration dates Birds | 85 | 35.71 | 150 | 63.03 | 3 | 1.26 | 15.7593 | 0.0274* | 0.9158 | 0.9691 | |  |
|  |  | Change reproduction dates Birds | 178 | 74.79 | 56 | 23.53 | 4 | 1.68 | 4.7416 | 0.6915 | 0.6306 | 0.9866 | |  |
|  |  | Change activity periods Insects | 62 | 26.05 | 176 | 73.95 | 0 | 0.00 | 8.6445 | 0.2792 | 2.6304 | 0.7567 | |  |
|  |  | Change migration dates Insects | 2 | 0.84 | 2 | 0.84 | 234 | 98.32 | 6.0958 | 0.5286 | 3.7434 | 0.5869 | |  |
|  | *Terrestrial Wild fauna Phenology* | | **23** | **9.66** | **215** | **90.34** | **0** | **0** | **11.9027** | **0.1038** | **2.4035** | **0.7910** | |  |
| **TERRESTRIAL WILD FLORA** | Wild flora Abundance | Change abundance Riverside trees | 36 | 15.13 | 202 | 84.87 | 0 | 0.00 | 11.2378 | 0.1286 | 2.2448 | 0.8143 | |  |
|  | *Wild flora Abundance* | | **36** | **15.13** | **202** | **84.87** | **0** | **0.00** | **11.2378** | **0.1286** | **2.2448** | **0.8143** | |  |
|  | Wild flora Distribution (plants-shrubs-trees) | Change altitudinal distribution Wild plants | 195 | 81.93 | 35 | 14.71 | 8 | 3.36 | 14.9330 | 0.0369* | 7.3985 | 0.1926 | |  |
|  | *Wild flora Distribution* | | **195** | **81.93** | **35** | **14.71** | **8** | **3.36** | **14.9330** | **0.0369*** | **7.3985** | **0.1926** | |  |
|  | Wild flora Disease/ pest/ mortality (plants-shrubs-trees) | Change abundance of diseases/plagues Wild plants | 41 | 17.23 | 195 | 81.93 | 2 | 0.84 | 10.6509 | 0.1546 | 3.9504 | 0.5566 | |  |
|  | *Wild flora Disease/ pest/ mortality* | | **41** | **17.23** | **195** | **81.93** | **2** | **0.84** | **10.6509** | **0.1546** | **3.9504** | **0.5566** | |  |
|  | Wild flora Phenology (plants-shrubs-trees) | Change flowering dates Wild plants | 84 | 35.29 | 153 | 64.29 | 1 | 0.42 | 11.0196 | 0.1378 | 2.7728 | 0.7350 | |  |
|  |  | Change germination dates NTFP* | 110 | 46.22 | 126 | 52.94 | 2 | 0.84 | 21.0241 | 0.0037* | 1.2993 | 0.9350 | |  |
|  | *Wild flora Phenology* | | **52** | **21.85** | **186** | **78.15** | **0** | **0** | **21.5669** | **0.0030*** | **1.5095** | **0.9120** | |  |
|  | Wild flora Productivity and Quality (plants-shrubs-trees) | Change growing patterns Wild plants | 100 | 42.02 | 135 | 56.72 | 3 | 1.26 | 21.7914 | 0.0028* | 3.9784 | 0.5525 | |  |
|  |  | Change abundance of NTFP* | 56 | 23.53 | 182 | 76.47 | 0 | 0.00 | 5.7103 | 0.5740 | 8.6524 | 0.1238 | |  |
|  |  | Change quality of NTFP* | 206 | 86.55 | 32 | 13.45 | 0 | 0.00 | 5.8861 | 0.5531 | 4.2366 | 0.5159 | |  |
|  | *Wild flora Productivity and Quality* | | **27** | **11.34** | **211** | **88.65** | **0** | **0** | **14.4649** | **0.0435*** | **6.5381** | **0.2573** | |  |
| **LAND DEGRADATION** | Wildfires | Change number of Fires | 120 | 50.42 | 118 | 49.58 | 0 | 0.00 | 62.6718 | 0.0000* | 4.1471 | 0.5284 | |  |
|  | *Wildfires* | | **120** | **50.42** | **118** | **49.58** | **0** | **0.00** | **62.6718** | **0.0000*** | **4.1471** | **0.5284** | |  |
| Human system | | | | | | | | | | | | | |  |
| **CULTIVATED SPP** | Cultivated spp productivity and quality | Change agricultural production | 55 | 23.11 | 177 | 74.37 | 6 | 2.52 | 16.0966 | 0.0242* | 7.9603 | 0.1584 | |  |
|  |  | Change growing patterns Crops | 128 | 53.78 | 103 | 43.28 | 7 | 2.94 | 20.7014 | 0.0042* | 8.6879 | 0.1222 | |  |
|  | *Cultivated spp productivity and quality* | | **44** | **18.49** | **188** | **78.99** | **6** | **2.52** | **26.6770** | **0.0004*** | **11.1797** | **0.0479*** | |  |
|  | Cultivated spp disease/ pest/ mortality | Change abundance of plagues in Crops (insects/nematodes) | 19 | 7.98 | 214 | 89.92 | 5 | 2.10 | 18.9171 | 0.0085* | 2.6277 | 0.7571 | |  |
|  |  | Change abundance of diseases in Crops (viruses/bacteria) | 22 | 9.24 | 211 | 88.66 | 5 | 2.10 | 23.4700 | 0.0014* | 3.1910 | 0.6706 | |  |
|  |  | Change percentage of damaged/mortality in Crops | 39 | 16.39 | 193 | 81.09 | 6 | 2.52 | 12.3169 | 0.0906 | 3.5346 | 0.6182 | |  |
|  | *Cultivated spp disease/ pest/ mortality* | | **7** | **2.94** | **226** | **94.9** | **5** | **2.10** | **16.1105** | **0.0241*** | **3.6900** | **0.5949** | |  |
|  | Cultivated spp Phenology and reproduction | Change germination/flowering/ maturation dates Crops | 56 | 23.53 | 176 | 73.95 | 6 | 2.52 | 8.6571 | 0.2782 | 1.9976 | 0.8495 | |  |
|  |  | Change altitudinal distribution Crops | 107 | 44.96 | 126 | 52.94 | 5 | 2.10 | 72.6342 | 0.0000* | 2.1906 | 0.8222 | |  |
|  | *Cultivated spp Phenology and reproduction* | | **29** | **12.18** | **204** | **85.71** | **5** | **2.10** | **38.3777** | **0.0000*** | **1.7889** | **0.8775** | |  |
| **PASTURES AND GRASSLAND** | Pasture availability and productivity | Change Pasture availability throughout the year | 5 | 2.10 | 109 | 45.80 | 124 | 52.10 | 10.5163 | 0.1612 | 114.1420 | 0.0000* | |  |
|  |  | Change amount of extra feed in barn for Livestock | 5 | 2.10 | 111 | 46.64 | 122 | 51.26 | 23.2248 | 0.0016* | 110.5463 | 0.0000* | |  |
|  | *Pasture availability and productivity* | | **1** | **0.42** | **117** | **49.16** | **120** | **50.42** | **17.1771** | **0.0163*** | **118.9435** | **0.0000*** | |  |
|  | Pasture spp composition, distribution, and quality | Change Pasture quality | 51 | 21.43 | 62 | 26.05 | 125 | 52.52 | 9.7128 | 0.2054 | 72.1059 | 0.0000* | |  |
|  | *Pasture spp composition, distribution, and quality* | | **51** | **21.43** | **62** | **26.05** | **125** | **52.52** | **9.7128** | **0.2054** | **72.1059** | **0.0000*** | |  |
| **LIVESTOCK** | Livestock productivity and quality | Change production by Livestock head | 31 | 13.03 | 80 | 33.61 | 127 | 53.36 | 8.6890 | 0.2758 | 78.1098 | 0.0000* | |  |
|  |  | Change quality raw material of Livestock | 79 | 33.19 | 32 | 13.45 | 127 | 53.36 | 11.8371 | 0.1060 | 18.9236 | 0.0020* | |  |
|  |  | Change honey production by Hive | 9 | 3.78 | 28 | 11.76 | 201 | 84.45 | 7.3325 | 0.3951 | 149.0778 | 0.0000* | |  |
|  | *Livestock productivity and quality* | | **23** | **9.66** | **108** | **45.38** | **107** | **44.96** | **6.9140** | **0.4379** | **71.1905** | **0.0000*** | |  |
|  | Livestock disease/ pest/ mortality | Change abundance of diseases/abortions/dehydration in Livestock | 18 | 7.56 | 93 | 39.08 | 127 | 53.36 | 9.3752 | 0.2268 | 67.3208 | 0.0000* | |  |
|  |  | Change abundance of diseases/parasites/predators in Bees | 1 | 0.42 | 38 | 15.97 | 199 | 83.61 | 8.1311 | 0.3212 | 174.1347 | 0.0000* | |  |
|  |  | Change percentage of death Bees per Hive | 6 | 2.52 | 29 | 12.18 | 203 | 85.29 | 7.5792 | 0.3712 | 171.3836 | 0.0000* | |  |
|  |  | Change frequency of mutations/deformities in Bees | 13 | 5.46 | 21 | 8.82 | 204 | 85.71 | 10.5889 | 0.1576 | 153.1498 | 0.0000* | |  |
|  | *Livestock disease/ pest/ mortality* | | **18** | **7.56** | **123** | **51.68** | **97** | **40.76** | **5.2122** | **0.6341** | **112.6890** | **0.0000*** | |  |
|  | Livestock phenology | Change unusual behaviour in Livestock | 83 | 34.87 | 27 | 11.34 | 128 | 53.78 | 2.0764 | 0.9555 | 44.6362 | 0.0000* | |  |
|  |  | Change altitudinal distribution of livestock | 68 | 28.57 | 34 | 14.29 | 136 | 57.14 | 9.9369 | 0.1922 | 36.4035 | 0.0000* | |  |
|  |  | Change Swarm grouping and Bee breeding dates | 15 | 6.30 | 19 | 7.98 | 204 | 85.71 | 4.7109 | 0.6952 | 121.3343 | 0.0000* | |  |
|  | *Livestock phenology* | | **65** | **27.31** | **64** | **26.89** | **109** | **45.79** | **1.9210** | **0.9641** | **57.3497** | **0.0000*** | |  |

*NTFP: Non timber forest products
