# Supplementary material for: Local reports of climate change impacts in Sierra Nevada, Spain: sociodemographic and geographical patterns
Source: Reg Environ Change. 2022 Dec 16;23(1):14. doi: 10.1007/s10113-022-01981-5 (PMC9758096; doi:10.1007/s10113-022-01981-5)
Supplement: Supplementary file 2 — Supplementary file2 (DOCX 22 KB) [file 10113_2022_1981_MOESM2_ESM.docx]

**Local reports of climate change impacts in Sierra Nevada, Spain: Sociodemographic and geographical patterns**

**Regional Environmental Change**

David García-del-Amo^*^; Peter Graham Mortyn; Victoria Reyes-García

* David.Garcia.delAmo@uab.cat

Institut de Ciència I Tecnologia Ambientals, Universitat Autònoma de Barcelona, Columnes S/N. Building ICTA-IPC(Z) UAB Campus, 08193, Bellaterra - Barcelona, Spain

Department of Geography, Universitat Autònoma de Barcelona, 08193, Bellaterra - Barcelona, Spain,

Online Resource 2

|  | LICCI INDEX CORE MODEL | LICCI INDEX  Excluded  Women | LICCI INDEX Excluded  Under 40 | LICCI INDEX Altitude | LICCI INDEX  Altitude Range |
| --- | --- | --- | --- | --- | --- |
|  |  |  |  |  |  |
| Profession (*others* omitted category) | . | . | . | . | . |
|  |  |  |  |  |  |
| Farmers | 0.0170 | 0.0086 | 0.0280 | 0.0124 | 0.0136 |
|  | (0.0267) | (0.0279) | (0.0276) | (0.0264) | (0.0264) |
| Shepherds | 0.1028*** | 0.0814** | 0.1150*** | 0.0863*** | 0.0991*** |
|  | (0.0317) | (0.0333) | (0.0321) | (0.0305) | (0.0309) |
| Ranchers | 0.0793 | 0.0691 | 0.0810 | 0.0680 | 0.0721 |
|  | (0.0447) | (0.0456) | (0.0452) | (0.0433) | (0.0433) |
| Beekeepers | 0.0515 | 0.0311 | 0.0604 | 0.0427 | 0.0448 |
|  | (0.0328) | (0.0350) | (0.0336) | (0.0321) | (0.0322) |
| Agricultural ranchers | 0.0935*** | 0.0905** | 0.0845** | 0.0917*** | 0.0816** |
|  | (0.0358) | (0.0372) | (0.0368) | (0.0350) | (0.0352) |
| Age | 0.0221*** | 0.0222*** | 0.0139 | 0.0203*** | 0.0199*** |
|  | (0.0064) | (0.0065) | (0.0089) | (0.0063) | (0.0063) |
| Age^2^ | -0.0002*** | -0.0002*** | -0.0001 | -0.0002*** | -0.0002*** |
|  | (0.0001) | (0.0001) | (0.0001) | (0.0001) | (0.0001) |
| Female | -0.0272 |  | -0.0358 | -0.0131 | -0.0193 |
|  | (0.0383) |  | (0.0395) | (0.0374) | (0.0375) |
| Schooling (*Bachelor or higher school completed* omitted category) | . | . | . | . | . |
| No schooling | 0.0954** | 0.1133** | 0.0938** | 0.1285*** | 0.1179*** |
|  | (0.0436) | (0.0454) | (0.0462) | (0.0425) | (0.0428) |
| Primary school completed | 0.0890** | 0.0980** | 0.0867** | 0.1157*** | 0.1109*** |
|  | (0.0366) | (0.0388) | (0.0396) | (0.0354) | (0.0356) |
| Secondary or vocational school completed | 0.0671 | 0.0807** | 0.0642 | 0.0795** | 0.0803** |
|  | (0.0363) | (0.0382) | (0.0398) | (0.0353) | (0.0354) |
| Parents born in Sierra Nevada | -0.1042** | -0.0951 | -0.0996 | -0.1258** | -0.1238** |
|  | (0.0514) | (0.0520) | (0.0518) | (0.0503) | (0.0504) |
| Grandparents born in Sierra Nevada | 0.0509** | 0.0522** | 0.0467 | 0.0611** | 0.0576** |
|  | (0.0246) | (0.0250) | (0.0246) | (0.0240) | (0.0240) |
| Years of experience agropastoral act. | -0.0016 | -0.0016 | -0.0018 | -0.0011 | -0.0014 |
|  | (0.0011) | (0.0012) | (0.0011) | (0.0011) | (0.0011) |
| Number of activities in nature | 0.0422*** | 0.0376*** | 0.0376*** | 0.0466*** | 0.0461*** |
|  | (0.0099) | (0.0101) | (0.0101) | (0.0096) | (0.0096) |
| Zone (*Marquesado del Cenete* omitted category) | . | . | . | . | . |
|  |  |  |  |  |  |
| Zone Granada´s surroundings | 0.0777** | 0.0820** | 0.0656 |  |  |
|  | (0.0378) | (0.0408) | (0.0389) |  |  |
| Zone Lanjarón and Lecrín Valley | 0.0034 | 0.0052 | 0.0026 |  |  |
|  | (0.0406) | (0.0426) | (0.0409) |  |  |
| Zone Poqueira Ravine | 0.0505 | 0.0597 | 0.0488 |  |  |
|  | (0.0390) | (0.0413) | (0.0397) |  |  |
| Zone Trevélez and Bérchules Ravines | 0.1009*** | 0.1316*** | 0.1076*** |  |  |
|  | (0.0386) | (0.0410) | (0.0398) |  |  |
| Zone Ohanes Ravine | 0.1071*** | 0.1117*** | 0.1116*** |  |  |
|  | (0.0390) | (0.0423) | (0.0394) |  |  |
| Zone Nacimiento Valley | 0.0438 | 0.0512 | 0.0566 |  |  |
|  | (0.0397) | (0.0417) | (0.0402) |  |  |
| Zone La Calahorra Valley | 0.0146 | 0.0273 | 0.0096 |  |  |
|  | (0.0392) | (0.0410) | (0.0401) |  |  |
| Altitude |  |  |  | -0.0000 |  |
|  |  |  |  | (0.0000) |  |
| Altitude range (*under 800 MSL* omitted category) |  |  |  |  | . |
|  |  |  |  |  |  |
| Altitude range (from 800 to 1049 MSL) |  |  |  |  | 0.0032 |
|  |  |  |  |  | (0.0257) |
| Altitude range (from 1050 to 1299 MSL) |  |  |  |  | -0.0351 |
|  |  |  |  |  | (0.0251) |
| Altitude range (over 1300 MSL) |  |  |  |  | 0.0444 |
|  |  |  |  |  | (0.0325) |
| _cons | 3.0705*** | 3.0544*** | 3.3588*** | 3.1569*** | 3.1683*** |
|  | (0.1918) | (0.1953) | (0.2764) | (0.1968) | (0.1912) |
| *N* | 238 | 222 | 225 | 238 | 238 |
| Standard errors in parentheses ** *p* < 0.05, *** *p* < 0.01 | | | | | |
